# Comparing the complications of laparoscopically performed simple, radical and donor nephrectomy

**DOI:** 10.3906/sag-1910-120

**Published:** 2020-06-23

**Authors:** Erkan ÖLÇÜCÜOĞLU

**Affiliations:** 1 Department of Urology, University of Health Sciences, Ankara City Hospital, Ankara Turkey

**Keywords:** Complications, laparoscopy, nephrectomy, kidney

## Abstract

**Bacground/aim:**

The aim of this study was to compare the complications of laparoscopic simple, radical and donor nephrectomies performed in a single center.

**Materials and methods:**

The study was conducted on 392 patients who underwent laparoscopic nephrectomy in University of Health Sciences, Ankara Türkiye Yüksek İhtisas Training and Research Hospital between January 1, 2008 and January 30, 2019. Clinical and laboratory parameters were recorded. Postoperative complications were recorded and graded as per Clavien-Dindo classification (CDC). All analyses were performed on SPSS v21.0 (IBM Corp., Armonk, NY, USA).

**Results:**

The mean age of the patients was 49.13 ± 15.45 years. The frequency of comorbidities and ASA scores were significantly higher in the laparoscopic radical nephrectomy (LRN) group than in the other groups (P < 0.001). Amount of bleeding was significantly lower in the laparoscopic donor nephrectomy (LDN) group compared to the other groups (P < 0.001). Classification of complications according to CDC showed that complications occurred in 17.01% (n = 25) of the LRN group, 7.02% (n = 12) of the laparoscopic simple nephrectomy (LSN) group, and 2.70% (n = 2) of the LDN group. Length of stay in hospital was significantly higher in the LRN group than in the LSN group (P < 0.001).

**Conclusion:**

In this study, the frequency of complications in LRN procedures was found to be higher than the LSN and LDN procedures. Patients with LRN may have more adverse health conditions before the operation. Considering the results of this study, variables such as patient and hospital characteristics, surgeon experience and skills should be evaluated in future studies. In addition, it is important to determine the frequency of complications using a standardized classification in order to enable correct interpretation of results.

## 1. Introduction

Nephrectomy, which is a common intervention in urology practice, can be performed in various ways. The first is the removal of a kidney that has lost function due to benign causes (simple nephrectomy), the second is the removal of a kidney due to malignant causes (radical nephrectomy) and the third is renal removal performed for the purpose of renal donation (donor nephrectomy) [1]. Laparoscopic approaches to all 3 of these nephrectomies have been accepted in urology clinical practice. In addition to the possibility of surgical problems, differences in the expected and desired outcomes of each approach may also affect the risk of postop complications [2]. Assessing and predicting these possibilities increase our understanding of the procedures used, and may reduce the frequency of unwanted outcomes.

The American Society of Anesthesiologists (ASA) grade classification, which is a simple system that shows preop physical status, is used to predict preoperative risk in patients who are to undergo nephrectomy [3]. In order to compare postoperative complications between centers and studies, various classification methods are utilized. Among these classifications, the Clavien-Dindo classification (CDC) which is a modified version of the classification system proposed by Clavien, is widely preferred in the classification of complications after many surgical interventions [4]. The European Association of Urology also recommends the use of CDC in patients undergoing nephrectomy [5]. In this system, the complications are divided into 5 grades that increase according to their severity [4,6].

Complications such as bleeding, vascular injuries of the liver, intestine and other important vessels, pain, apnea, emphysema, ileus, and wound infections, can be seen in laparoscopic urological procedures [7]. Potential preoperative risks (such as hypertension, diabetes mellitus, chronic obstructive pulmonary disease), as well as increased body mass index (BMI), poor renal function and abnormalities in vascular structure increase the risk of complications [8]. Studies evaluating the results of laparoscopic nephrectomy methods report varying degrees and frequencies of complications. The lack of standardization of complication detection between studies may be the cause of these differences. It is also apparent that the number of studies with a sufficient number of patients from a single-center that would enable accurate comparison of laparoscopic nephrectomy techniques is very low. Data regarding the complications of these techniques is crucial for the selection of appropriate surgical technique and the determination of preoperative precautions.

It is well known that surgical techniques may significantly affect postoperative complications. The aim of this study was to compare the complications of simple, radical and donor nephrectomies performed in a single center.

## 2. Material and methods

The study was conducted on 392 patients who underwent laparoscopic nephrectomy in University of Health Sciences, Ankara Türkiye Yüksek İhtisas Training and Research Hospital between January 1, 2008 and January 30, 2019. A total of 171 laparoscopic simple nephrectomy (LSN), 147 laparoscopic radical nephrectomy (LRN), 74 laparoscopic donor nephrectomy (LDN) cases were included in the study. Patients whose medical records were not complete were excluded from the study. Ethics committee approval was received from the ethics committee of University of Health Sciences, Ankara Türkiye Yüksek İhtisas Training and Research Hospital (Approval number: 29620911-929). The transperitoneal laparoscopic approach was used in all cases.

### 2.1. Measurements

The patients’ age, sex, weight, height, BMI, comorbidity, ASA scores, history of surgery, and nephrectomy characteristics were recorded and evaluated. Preoperative and postoperative hemoglobin, hematocrit and creatinine values, duration of the operation, amount of bleeding, blood transfusion, conversion (if necessary), and the length of stay at hospital were also recorded. Postoperative complications were recorded and graded as per CDC.

### 2.2. Statistical analysis

All analyses were performed on SPSS v21.0 (IBM Corp., Armonk, NY, USA). The normality of distribution of quantitative values was checked with the Kolmogorov-Smirnov test with Lilliefors correction. Quantitative data is given as mean ± standard deviation or median (minimum-maximum) with regard to normality and qualitative parameters are given as frequency (percentage). Age was analyzed with the 1-way analysis of variances (ANOVA) test and pairwise comparisons of age were performed with the Tamhane test (variances were nonhomogenous). Hemoglobin and hematocrit value comparisons were analyzed with 2-way repeated measures ANOVA. Nonnormally distributed variables were analyzed with the Kruskal-Wallis test. Analysis of creatinine levels were performed with Wilcoxon signed ranks test for repeated measurements and between-group comparisons of creatinine were performed by analyzing differences between measurements with the Kruskal-Wallis test. If pairwise comparisons were needed, the Bonferroni correction method was used. Categorical variables were analyzed with chi-square tests. P values equal or lower than 0.05 were accepted to demonstrate statistical significance.

## 3. Results

The mean age of the patients was 49.13 ± 15.45 years and those in the LRN group were found to be significantly older than the other patients (P < 0.001). Patients in the LRN group were also significantly taller (P = 0.003) and had a higher BMI (P = 0.016) than those in the LDN group. The frequency of comorbidities and ASA scores were significantly higher in the LRN group than in the other groups, and were lowest in the LDN group (P < 0.001). History of surgery was significantly less frequent in the LDN group than the other groups (Table 1).

**Table 1:** Summary of patients’ characteristics according to type of nephrectomy.

	Simple (n = 171)	Radical (n = 147)	Donor (n = 74)	P
Age	44.54 ± 16.45a	57.61 ± 11.60b	42.89 ± 12.05a	<0.001
Sex				
Male	91 (53.22%)a	103 (70.07%)b	35 (47.30%)a	0.001
Female	80 (46.78%)	44 (29.93%)	39 (52.70%)	
Weight (kg)	72.5 (34–130)ab	79 (49–120)a	70 (50–100)b	0.003
Height (cm)	165 (138–192)a	170 (145–190)b	167 (140–184)ab	0.004
BMI (kg/m2)	26.6 (14.2–46.9)ab	27.6 (17–41.5)a	25.3 (17.9–45.4)b	0.016
Comorbidity	77 (45.03%)a	99 (67.35%)b	1 (1.35%)c	<0.001
Diabetes mellitus	15 (8.77%)	32 (21.77%)	0 (0.00%)	
Hypertension	52 (30.41%)	73 (49.66%)	0 (0.00%)	
Coronary artery disease	9 (5.26%)	30 (20.41%)	0 (0.00%)	
COPD	7 (4.09%)	8 (5.44%)	1 (1.35%)	
Cerebrovascular disease	1 (0.58%)	1 (0.68%)	0 (0.00%)	
Chronic renal failure	11 (6.43%)	2 (1.36%)	0 (0.00%)	
Epilepsy	2 (1.17%)	0 (0.00%)	0 (0.00%)	
ASA scores				
I	65 (38.01%)a	33 (22.45%)b	61 (82.43%)c	<0.001
II	89 (52.05%)	78 (53.06%)	13 (17.57%)	
III	16 (9.36%)	35 (23.81%)	0 (0.00%)	
IV	1 (0.58%)	1 (0.68%)	0 (0.00%)	
History of surgery	45 (26.32%)a	41 (27.89%)a	5 (6.76%)b	<0.001
Side				
Right	61 (35.67%)a	58 (39.46%)a	6 (8.11%)b	<0.001
Left	104 (60.82%)	89 (60.54%)	68 (91.89%)	
Bilateral	6 (3.51%)	0 (0.00%)	0 (0.00%)	

Data given as mean ± standard deviation or median (minimum-maximum) for continuous variables with regard to normality of distribution and as frequency (percentage) for categorical variables. ASA: American Society of Anesthesiologists, BMI: Body mass index, COPD: Chronic obstructive pulmonary disease.Same letters denote the lack of statistical difference between respective groups.

Amount of bleeding was significantly lower in the LDN group compared to the other groups (P < 0.001). Classification of complications according to CDC showed that complications occurred in 17.01% (n = 25) of the LRN group, 7.02% (n = 12) of the LSN group, and 2.70% (n = 2) of the LDN group (Figure). In the LSN group, there were 8 cases with grade 1, 3 cases with grade 2, and 1 case with grade 4 complications. In the LRN group, 20 complications were grade 1, 4 were grade 2 and 1 was grade 3. In the LDN group, 1 patient had grade 1 and the other had grade 2 complications. Since the number of patients per group was not sufficient, no comparisons were performed to determine differences regarding the CDC grade of complications. Length of stay in hospital was significantly higher in the LRN group than in the LSN group (P < 0.001). There was no difference between hemoglobin and hematocrit values with regard to type of nephrectomy; whereas postop values in each group were significantly lower than preop values (P < 0.001). The amount of creatinine change in the LSN group was less than the other 2 groups. Also, there was no difference between preoperative and postoperative values in this group (Table 2).

**Figure:**
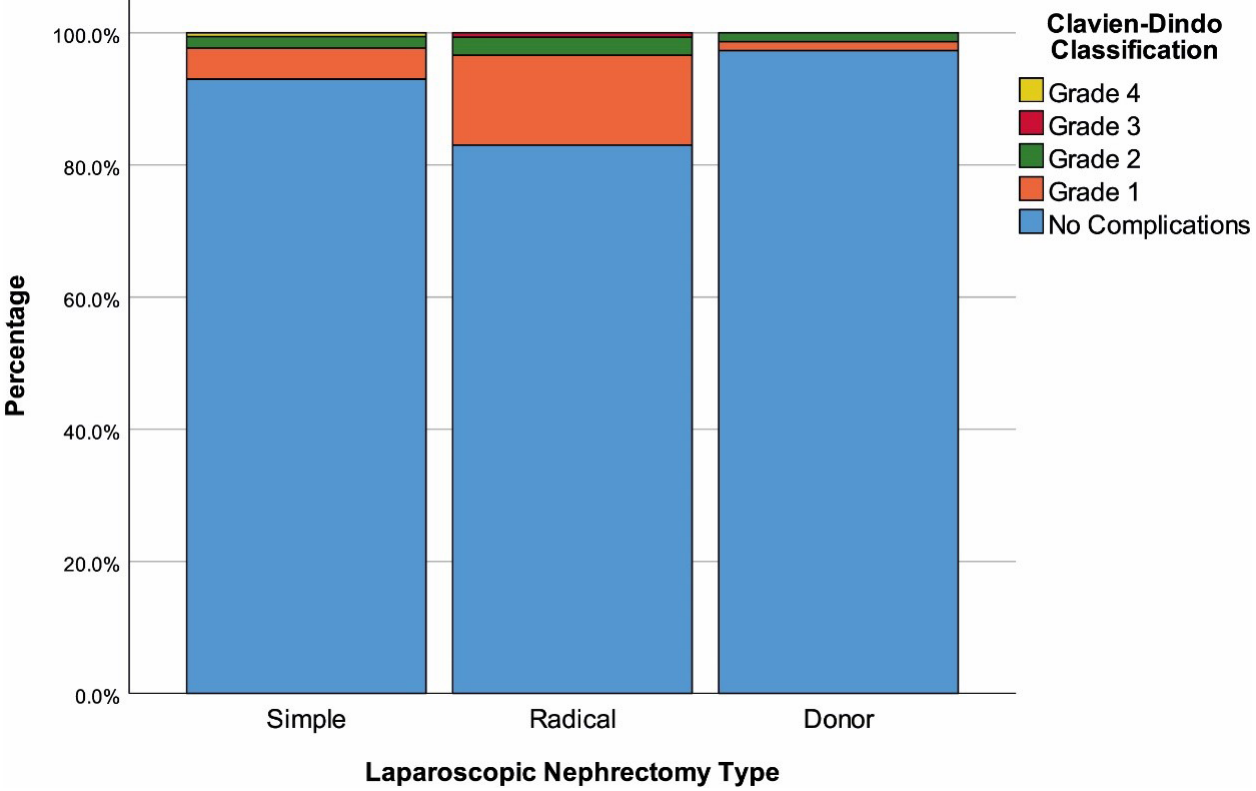
Distribution of complications according to type of nephrectomy.

**Table 2:** Summary and comparisons of procedure-related characteristics according to nephrectomy type.

	Simple (n = 171)	Radical (n = 147)	Donor (n = 74)	P
Duration of operation	110 (50–300)a	125 (40–420)b	130 (90–240)b	<0.001
Amount of bleeding	50 (0–400)a	50 (0–3200)a	35 (0–250)b	<0.001
Blood transfusion	1 (0.59%)	6 (4.08%)	2 (2.70%)	0.114
Conversion				
No	167 (97.66%)	143 (97.28%)	74 (100.00%)	0.376
Yes	4 (2.34%)	4 (2.72%)	0 (0.00%)	
Clavien-Dindo classification				
No complications	159 (92.98%)a	122 (82.99%)b	72 (97.30%)a	0.017
Grade 1	8 (4.68%)	20 (13.61%)	1 (1.35%)	
Grade 2	3 (1.75%)	4 (2.72%)	1 (1.35%)	
Grade 3	0 (0.00%)	1 (0.68%)	0 (0.00%)	
Grade 4	1 (0.58%)	0 (0.00%)	0 (0.00%)	
Grade 5	0 (0.00%)	0 (0.00%)	0 (0.00%)	
Length of stay in hospital	3 (0–15)a	4 (2–10)b	3 (1–7)ab	<0.001
Hemoglobin				
Preop	13.64 ± 2.01	13.63 ± 1.98	14.08 ± 1.68	0.781
Postop	12.25 ± 1.97	12.28 ± 1.74	12.81 ± 1.91	
P (within groups)	<0.001	<0.001	<0.001	
Hematocrit				
Preop	41.02 ± 5.82	41.33 ± 5.58	42.76 ± 4.80	0.891
Postop	36.82 ± 5.70	37.31 ± 5.06	38.70 ± 5.45	
P (within groups)	<0.001	<0.001	<0.001	
Creatinine				
Preop	1.01 (0.59–6.62)	0.99 (0.55–5.10)	0.77 (0.39–1.06)	<0.001
Postop	1.02 (0.47–8.73)a	1.19 (0.57–16.00)b	0.99 (0.54–1.94)b	
P (within groups)	0.301	<0.001	<0.001	

Data given as mean ± standard deviation or median (minimum-maximum) for continuous variables regarding normality and frequency (percentage) for categorical variables.Same letters denote groups do not differ significantly from each other.

## 4. Discussion

Although laparoscopic surgeries have become preferred in many centers due to their safety and efficacy, these procedures may still cause significant adverse events. The degree and frequency of postoperative complications reportedly show significant variance depending on the type of transperitoneal laparoscopic nephrectomy. In this study comparing the complications of 3 different laparoscopic nephrectomy approaches according to CDC, it was found that complication frequency was significantly higher in the LRN procedure compared to the other procedures, and most of the complications (80%) were grade 1. These patients were also older, had a higher frequency of comorbidities, and had higher ASA scores.

In the literature, complication rates are reported to vary between 4.4% and 25.8% in laparoscopic urological procedures [9–12]. When the studies comparing the results of the same techniques with our study were examined, we found that Permpongkosol et al. reported LSN, LDN and LRN complication rates according to CDC, as 10.2%, 23.5%, and 13.7%, respectively. Although overall complications were higher with LDN, it was found that major complications were more common in LRN, whereas minor complications were more common in LDN. They also reported that the length of hospital stay correlated with the incidence of complications [13]. Kim et al. reported that the frequency of complications was not different between techniques in their single-center study comparing the complications of all 3 nephrectomy types. They stated that obesity did not affect the risk, and increased ASA score significantly increased the risk of complications. Although there was no significant difference between the groups, it was reported that the highest frequency was with LDN (15.2%), followed by LRN (13.7%) and LSN (10%) [14]. In a meta-analysis, Pareek et al. evaluated 56 studies (1995 to 2004) examining the complications of laparoscopic renal surgery and including at least 20 adult cases. LSN was found to be associated with a 13.7% frequency of major complications, while frequencies were 10.7% for LRN and 10.6% for LDN [15]. These results demonstrate a significant difference from our findings, which may be explained by several factors: the fact that the frequency of complications in those studies were not determined by a standardized procedure, the possibility that minor complications may have been overlooked in the absence of definitive criteria, and the changes throughout the years that may have influenced the quality of patient care and surgical procedures. In a review by Fowler et al., the incidence of nephrectomy was found to increase while complication rates decreased in the United Kingdom over the years. It was reported that the incidence of postoperative complications after LRN operations between 2002 and 2012 was higher than LSN (11.7% vs. 8.3%). In addition, the length of hospital stay was reported to be longer in the LRN group (4 days vs. 3 days) [16]. Many studies focusing on the results of laparoscopic nephrectomy techniques have been conducted and different results are reported in many. The results of the studies using CDC, a standard classification tool for complications, are similar to our results. The use of different methods to identify complications makes the comparisons between studies difficult and limits feasible comments on the results.

The undesirable results of procedures were evaluated with the frequency of complications in the current study. Similar results have been reported in studies evaluating different postop outcomes of these surgical techniques. Verma et al. compared renal functions after LRN, LSN and LDN. Consistent with our study, they reported that patients undergoing LRN were older, had a higher frequency of comorbid diseases, and experienced a higher frequency and severity of chronic kidney disease postoperatively. The best results were reported in the LDN group [17]. In another study evaluating the effect of all 3 laparoscopic nephrectomy techniques on health-related quality of life, Wiesenthal et al. reported that patients undergoing LRN were significantly older than the other 2 groups. No significant postoperative complications were reported in patients undergoing LSN. Complications developed at similar frequencies after LRN and LDN, and the shortest hospital stay was reported in LSN (2.2 vs. 4.4 days) [18]. Postoperative undesirable outcomes after laparoscopic nephrectomy are consistent with our study and seem to mostly affect patients undergoing LRN.

Among the studies evaluating only LSN results, Hsiao and Pattaras reported the incidence of complications as 21.4%, [19], Manish Garg et al. reported that the incidence of complications was 25.8% and the mean length of hospital stay was 5.7 ± 3.36 days [12]. In our study, the frequency of complications (7.02%) and length of hospital stay (3 days) were lower in patients undergoing LSN than the results of these studies. Considering that the frequency of complications and the length of hospital stay may be correlated, it is rather evident that the shorter duration of hospital stay in our study is associated with the presence of fewer complications. Among the studies evaluating only LDN results, Treat et al. reported 7.9% (6.1% grade 1) complication frequency, and 1.37 (1–10) days of hospital stay [20], Schold et al. reported 7.9% complication frequency [21], Srivastava et al. reported 8.6% postoperative complication rate (the majority being grade 1), and 3.8 ± 10.5 days of hospital stay [22]. In studies evaluating the frequency of complications with different methods, an overall higher frequency of complications has been reported [23–25]. In our study, patients who underwent LDN were found to have the least complications (2.7%) and the median duration of hospitalization was 3 (1–7) days, similar to the literature. The low complication rate can be explained by the fact that patients have no previous surgical history, relatively low ASA levels, and relatively low BMI values. Among the studies evaluating only LRN results, a cohort study conducted by Gozen et al. in a high-capacity center reported the incidence of complications as 19.7% (5.1% grade 1, 7.6% grade 2) [26], while in another study, Permpongkosol et al. reported 20% postop complication rate [27]. In our study, similar to the literature, a total of 17% postop complications were observed after LRN, mostly grade 1 according to CDC. Abbou et al. evaluated the incidence of retroperitoneal LRN complications using an older version of CDC and reported complications in 8% of LRN patients [28]. We thought that this result was due to different classification of complications. Hospital characteristics, surgeon experience, and characteristics affecting the overall health of patients may have affected the results of the studies.

In addition to preoperative comorbidities, increased BMI, poor renal function and abnormalities in vascular structure have been shown to increase the risk of complications [22,29,30]. In our study, the incidence of comorbid diseases such as diabetes mellitus, hypertension and chronic obstructive pulmonary disease was found to be higher in LRN patients than other groups. BMI was also highest in the LRN group. Studies have shown that comorbidity and BMI increase the risk of postoperative complications, and the association of these conditions with age is also another factor that increases risk [22,31]. In our study, the effect of these variables on the complication score could not be examined. The fact that patients with significantly worse health characteristics were stratified to the LRN and partially to the LSN group may have led to selection bias which would render comparisons in this regard unfeasible. On the other hand, Arfi et al. examined the effect of obesity on the results of LSN and LRN operations, and stated that obesity did not affect the incidence of complications, but increased the duration of the operation [32]. Another finding that may explain the increased frequency of complications in the LRN group is that the ASA scores, which measure preoperative physical health status, were significantly higher in the LRN group than the other 2 groups. Studies have reported that more experience and higher surgical skill reduces the risk of complications [22,33]. Similarly, as more nephrectomy operations are performed in centrally located hospitals with more patient potential, surgical success may increase and the incidence of complications may decrease [34,35]. Since hospital characteristics and surgeons performing operations could not be standardized, the impact of the hospital and experience/skill differences between surgeons could not be evaluated in the current study.

The number of cases evaluated in our study is comparatively high when considering the studies in the literature. Although single centeredness limits generalizability, it also ensures that the procedures were performed at a similar standard. These are among the strengths of our study that increase the value of evidence. The characteristics of our hospital in comparison with other centers and the experience / skill differences between surgeons could not be evaluated. In addition, patients with conditions that increase the risk of complications (high BMI, age, ASA score, presence of comorbidity) were not equal in the groups, causing baseline differences. However, these differences are to be expected in the comparison of surgeries performed with different goals, and the majority of studies in this field demonstrate this weakness. These are among the limitations of our study.

In conclusion, laparoscopic procedures are preferred more frequently due to the lower frequency of complications and satisfactory results. Evaluation of the results of laparoscopic nephrectomies, which are widely used in urology practice, is crucial to increase the quality of surgeries. In this study, the frequency of complications in LRN procedures was found to be higher than the LSN and LDN procedures. Patients with LRN may have more adverse health conditions before the operation. Considering the results of this study, variables such as patient and hospital characteristics, surgeon experience and skills should be evaluated in future studies. In addition, it is important to determine the frequency of complications using a standardized classification in order to enable correct interpretation of results.

## Disclaimers/Conflicts of interest 

The authors have no conflict of interest and also declares that there is no funding for conducting to this study. We did not receive any funding source regarding the current study.
